# Early Introduction of Everolimus Immunosuppressive Regimen in Liver Transplantation with Extra-Anatomic Aortoiliac-Hepatic Arterial Graft Anastomosis

**DOI:** 10.1155/2014/493095

**Published:** 2014-09-21

**Authors:** Emanuele Felli, Giovanni Vennarecci, Marco Colasanti, Roberto Santoro, Edoardo de Werra, Andrea Scotti, Mirco Burocchi, Giovanni B. Levi Sandri, Alessandra Campanelli, Pasquale Lepiane, Giuseppe M. Ettorre

**Affiliations:** Digestive and Transplant Liver Surgery Unit, S. Camillo Hospital, Piazza Carlo Forlanini 1, 00151 Rome, Italy

## Abstract

Liver transplantation is the treatment of choice for patients with acute and chronic end-stage liver disease, when no other medical treatment is possible. Despite high rates of 1- to 5-year survival, long-term adverse effects of immunosuppressant agents remain of major concern. Current research and clinical efforts are made to develop immunosuppressant agents that minimize adverse effects along with a low rate of graft rejection. Tailoring immunosuppressive therapy to individual patients by the use of proliferation signal inhibitors seems to be the best way to minimize toxicity and increase efficacy. Recently everolimus has been introduced in clinical practice; among its adverse effects an increased incidence of arterial graft thrombosis in renal transplants, vascular anastomosis leakage, impaired wound healing, and thrombotic microangiopathy have been reported. We present the case of a 54-year-old patient submitted to liver transplantation for end-stage liver disease treated by an extra-anatomic aortoiliac-hepatic arterial graft anastomosis and early postoperative introduction of everolimus for acute renal failure. Postoperative period was characterized by two abdominal collections and reactivation of cytomegalovirus infection that were treated by percutaneous drainage and antiviral therapy, respectively; the patient is well after 8-month followup with patency of the arterial conduit and no leakage.

## 1. Introduction

Liver transplantation is the treatment of choice for patients with acute and chronic end-stage liver disease when no other medical treatment is possible [1]. Despite high rates of 1- to 3-year survival, major concerns remain over long-term adverse effects of immunosuppressant agents, such as diabetes, dyslipidemia, renal insufficiency, hypertension, and osteoporosis, all of which compromise quality of life and long-term patient survival [2–4]. The current clinical challenge is to develop regimens that maintain high rates of transplantation success while minimizing unwanted and harmful metabolic and other effects. It appears increasingly likely that efficacy and toxicity may be balanced by tailoring immunosuppressive therapy to individual patients by the use of proliferation signal inhibitors. These agents, which include the macrolide semisynthetic derivative of rapamycin, everolimus, appear to be well tolerated especially when used at lower dosages. Among side effects of everolimus episodic arterial graft thrombosis that has been described in renal transplant patients during the first postoperative month are vascular anastomosis leakage, impaired wound healing, and thrombotic microangiopathy. We report the case of a 54-year-old patient that was submitted to liver transplantation in our unit for HCV and alcohol abuse cirrhosis, with MELD score of 40 treated by an extra-anatomic aortoiliac-hepatic arterial graft anastomosis and early postoperative introduction of everolimus immunosuppressive regimen for acute renal failure.

## 2. Case Report

We present the case of a 54-year-old patient affected by HCV and alcohol abuse cirrhosis with end-stage liver disease with acute liver failure due to spontaneous bacterial peritonitis and MELD score of 40 submitted to our unit for orthotopic liver transplantation. In his past medical history we found blood hypertension controlled by medications, alcohol abuse, past heroin addiction, and chronic HCV infection.

**Figure 1:**
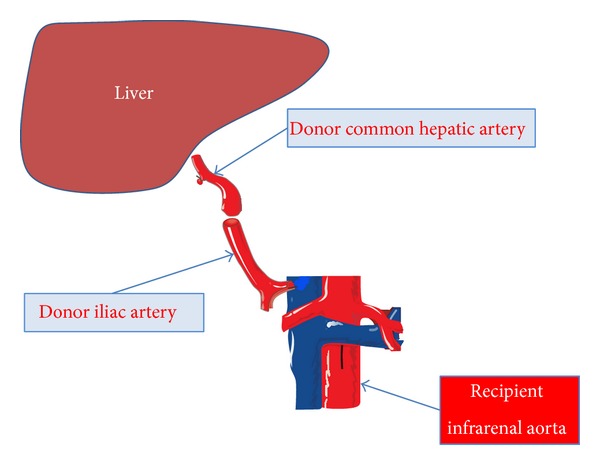
Schematic representation of the arterial conduit between the donor common hepatic artery and recipient infrarenal aorta.

The graft was procured by cadaveric donor. Cold ischemia time was 8 hours. Caval anastomosis was performed between the patient hepatic veins and the donor inferior vena cava; initially an end-to-end arterial anastomosis was performed between the donor celiac trunk and the patient common hepatic artery after the incision of a diaphragmatic median arcuate ligament; the biliary anastomosis was end-to-end protected by a T-Kehr tube. No temporary portocaval anastomosis was performed. Hemodynamic instability was present throughout the operation with consistent hypotension nonresponsive to medical treatment. Blood loss was 7000 mL with 5 packed red blood cells and 26 fresh frozen plasma transfused. Operative time was 10 hours. Arterial Doppler in the end of the operation showed a good flow with a RI index in the normal range. In the second postoperative day the patient was reoperated on for hemorrhagic shock secondary to hemoperitoneum. No evident cause of bleeding was found in the second operation. The final arterial Doppler was not satisfactory with a low flow and arterial graft hypopulsatility. Then, an extra-anatomic arterial by-pass using cadaveric iliac artery graft was performed with an end-to-end anastomosis between the common hepatic artery and the donor iliac artery in the proximal side and a side-to-end anastomosis between the distal abdominal aorta and the donor iliac artery (Figure 1). Arterial Doppler at the end of the second operation was normal. Early immunosuppression in our institution is based on the combination of tacrolimus, corticosteroids, and mycophenolate mofetil, and this regimen was applied to the presented patient. In the first postoperative week the patient developed acute renal insufficiency and so we decided to make an early switch to everolimus. Daily arterial Doppler showed normal flow, with good patency of the arterial graft. In the postoperative period two intra-abdominal collections developed, all drained percutaneously, along with cytomegalovirus reactivation treated by antiviral agents. After eight-month followup the patient is well with good patency of the arterial graft and normal liver function.

## 3. Discussion

Everolimus, a proliferation signal inhibitor, prevents allograft rejection in rodent and nonhuman primate models of allotransplantation. It exerts its immunosuppressive effect by inhibiting the proliferation and thus clonal expansion of antigen-activated T cells which is driven by T cell specific interleukins. Everolimus inhibits an intracellular signalling pathway which is triggered upon binding of these T cell growth factors to their respective receptors and which normally leads to cell proliferation. Its application in human transplantation has been already described; among its adverse effects arterial graft thrombosis has been reported in renal transplants within the first month of transplantation. In the phase III, randomised, controlled, open-label study (H2304) [5], reduced tacrolimus exposure and everolimus were administered to HCV positive and HCV negative patients starting approximately 4 weeks after transplantation, investigated versus standard tacrolimus exposure. A third arm in study H2304 with complete withdrawal of tacrolimus at 4 months after transplantation was terminated early because it has been associated with an increased risk of acute rejections. In the 12-month analysis, the incidence of the composite endpoint (biopsy proven acute rejection, graft loss, or death) was lower in everolimus + reduced tacrolimus arm compared to the tacrolimus control arm. The reduction in glomerular filtration rate was less substantial at 12 months and at 24 months. We decided to introduce everolimus in the patient immunosuppressive treatment in relation to the acute postoperative renal insufficiency, thus minimizing anticalcineurin renal damage. Impaired wound healing, thrombotic microangiopathy, vascular leakage, and intra-abdominal collections, especially lymphocele, have been described as adverse effects of everolimus treatment. In this case it is difficult to relate the two abdominal collections that appeared in the postoperative period to the use of everolimus because of the multiple operations and the intra- and postoperative complications. In conclusion we present the case of early introduction of everolimus treatment in the setting of a difficult liver transplantation that was not associated with arterial thrombosis, vascular leakage, wound healing alteration, and microangiopathy.
